# The Role of Single Nucleotide Polymorphisms in Beta-2 Adrenergic Receptors in the Severity of Obstructive Sleep Apnea Syndrome in Pediatric Patients

**DOI:** 10.7759/cureus.74477

**Published:** 2024-11-26

**Authors:** Marco Ferrari, Eleonora Sica, Francesca De Bernardi, Alessandra Luini, Massimiliano Legnaro, Luana Nosetti, Paolo Castelnuovo, Marco Cosentino, Franca Marino

**Affiliations:** 1 Department of Medicine and Surgery, University of Insubria, Varese, ITA; 2 Department of Pediatrics, University of Insubria, Varese, ITA; 3 Department of Otorhinolaryngology, University of Insubria, Varese, ITA

**Keywords:** apnea-hypopnea index (ahi), beta-2 adrenergic receptors, obstructive sleep apnea syndrome (osas), pediatrics patients, single nucleotide polymorphisms

## Abstract

Background: Obstructive sleep apnea syndrome (OSAS) is a chronic syndrome, affecting about 1%-5% of children. OSAS is characterized by increased resistance and collapse of the upper airways, with different degrees of severity requiring interventions ranging from lifestyle modifications to surgery. Sympathetic activity is increased in OSAS, and the reduction of disease symptoms, occurring after adenotonsillectomy, correlates with biomarkers indicating a reduced sympathetic response. The aim of this study is to explore the potential role of single nucleotide polymorphisms (SNPs) in the gene encoding β2-adrenergic receptors (ADRB2) as a biomarker for the early identification of pediatric OSAS patients at high risk of developing severe symptoms.

Materials and methods: In this exploratory genetic study, the frequencies of functional SNPs in ADRB2 within a cohort of pediatric patients were evaluated by using reverse transcription-polymerase chain reaction with TaqMan probes. The severity of OSAS was assayed by the apnea-hypopnea index (AHI).

Results: The rs1042713 SNP (GG genotype) in ADRB2 was more frequent in patients with severe OSAS compared to patients with mild/moderate OSAS.

Conclusions: The availability of genetic biomarkers for the early identification of patients at high risk of severe OSAS will help clinicians start personalized treatments, thus reducing morbidity associated with OSAS.

## Introduction

Obstructive Sleep Apnea Syndrome (OSAS) is a complex chronic syndrome that affects about 1%-5% of children and is characterized by increased resistance and collapse of the upper airways during sleep [1]. OSAS may have different degrees of severity, requiring interventions ranging from lifestyle modifications to surgery [2]. In children, undiagnosed and untreated OSAS can lead to significant complications, including behavioral problems, learning difficulties, cardiovascular issues, neurobehavioral challenges, and growth delays [3].

OSAS is a multifactorial disease that involves genetic, environmental, and developmental factors, including inflammatory processes [3,4]. Polymorphonuclear neutrophils (PMNs) are the first line of defense in upper airway inflammations and infections [5], and their number is increased in OSAS patients [6]. Another frequent occurrence in OSAS patients is increased production of catecholamines (CTs) and enhanced sympathoadrenergic activity [7].

CTs act on different adrenergic receptors, including β2-adrenergic receptors (ADRB2), which are G-protein-coupled receptors widely expressed in the respiratory system where they reduce upper airway smooth muscle tone and facilitate bronchi relaxation [8,9]. ADRB2 receptors are also present in circulating PMN, where they modulate key cellular functions such as reactive oxygen species (ROS) production, neutrophil extracellular trap production formation, and cell migration [5,10,11]. We recently reported that, in children with OSAS, adenotonsillectomy not only reduced OSAS symptoms but also improved the patient's inflammatory profile and reduced ADRB2 mRNA expression levels in PMN [12].

It is well known that single nucleotide polymorphisms (SNPs) in ADRB2 are able to modify receptor activity and expression [13,14]. Although some studies have evaluated the role of SNPs in ADRB2 in obstructive pulmonary diseases [15,16], so far no information exists on the role of SNPs in ADRB2 in pediatric OSAS patients. Building on our previous findings [12], this exploratory study aims to test the hypothesis that functional SNPs in ADRB2, previously associated with receptor expression [13,14], could serve as useful biomarkers for the early identification of pediatric OSAS patients at high risk of developing severe symptoms.

## Materials and methods

Study design and patients

This explorative study was conducted at the otorhinolaryngologist and pediatric departments of ASST Sette Laghi, Varese, Italy, from May 2019 to July 2021. The study included nine patients who had taken part in a previous investigation on pediatric patients with OSAS [12] and had provided informed consent for genotyping. Furthermore, nine new pediatric OSAS patients were consecutively enrolled over a 12-month period. The study was approved by the Insubria Ethics Committee (study number: 14/2019), and patients were enrolled after parents/tutors read and signed an informed consent form.

We enrolled subjects ranging from three to eight years of age, with a diagnosis of OSAS performed by NOX T3 (Nox Medical, Alpharetta, USA) [17] eligible for adenotonsillectomy. We excluded patients with other systemic diseases with facial malformations, showing recent episodes of upper airway inflammation, taking steroids, anti-leukotriene, antibiotics, and anti-histaminergic therapy in the last two weeks before surgery.

Demographic and clinical features, including inflammatory parameters such as Interleukin (IL)-6, IL-8, C-reactive protein (CRP), erythrocyte sedimentation rate (ESR), neutrophils, and eosinophils, were collected for each patient from medical records. ADRB2 mRNA expression levels in PMN were evaluated by real-time PCR according to published procedures [18].

OSAS severity was evaluated by the apnea-hypopnea index (AHI, i.e., the total episodes of apnea and hypopnea occurring per hour of sleep) [17,19]. AHI was obtained by NOX T3 polygraph. Collected data was then analyzed by dedicated software (Noxturnal software 3.0, NOX Medical). Based on the AHI score, patients were divided into two groups. Group one (severe OSAS grade) includes subjects with AHI > 10, and group two (mild/moderate OSAS grade) subjects with AHI ≤ 10 [20].

SNPs selection criteria and genotyping

We selected a panel of SNPs, giving priority to those with an expected frequency in Caucasian populations of at least 2%, with evidence of functional relevance in previous studies. Drawing from our previous experience [21-23], the choice of SNPs with high frequencies increases the probability of identifying potential differences in their frequency in relation to relevant phenotypic aspects, even within a relatively small patient cohort. Furthermore, selecting SNPs with well-established biological effects enhances the likelihood of their contribution to determining patients' phenotypes (Table 1).

**Table 1:** ADRB2 gene variants included in the study AF: allelic frequencies in Caucasian populations; SNP: single nucleotide polymorphism

SNP	Nucleotide change	Amino acid change	AF (%)	Allele functions
rs1042713	c.146G/A	Gly16Arg	42	Gly16 was associated with enhanced agonist-induced down-regulation [13,14].
rs1042714	c.179C/G	Gln27Glu	40	Glu27 was associated with reduced agonist-induced down-regulation [13].
rs1800888	c.491C/T	Thr164Ile	2	Ile164 was associated with lower agonist binding affinities [13].

A drop of 40 ul of blood from patients was obtained by a lancing device, and then genomic DNA was extracted by the Whatman FTA Elute Micro Card kit (Qiagen, Valencia, USA) as described by the manufacturer (https://it.vwr.com/store/product/en/7997552/fta-elute-cards-whatmantm?languageChanged=en). SNPs listed were identified by a pre-designed genotyping assay (ABI) using a TaqMan probe with a StepOne Real-Time PCR System (Applied Biosystems, Foster City, USA).

Statistical analysis

Data are shown as the mean ± standard deviation (SD) unless otherwise stated. The statistical significance of the differences between groups was assessed by the Mann-Whitney U-test. The χ2-test was used to assess the Hardy-Weinberg equilibrium. Genotype frequencies were analyzed by the χ2-test for trend (or by Fisher's exact test, as appropriate) or by analysis of variance (ANOVA) with a post-test for linear trend. The odds ratio (OR) with a 95% confidence interval (CI) was calculated using a recessive model (wild type/heterozygous versus homozygous).

Statistical analyses were performed using GraphPad Prism version 5.00 for Windows (San Diego, California, USA, www.graphpad.com).

## Results

Patients

The study enrolled 18 OSAS patients, 50% female, with an age (mean ± SD) of 5.2 ± 1.5 years, and overall AHI (mean ± SD) was 10.7 ± 7.1. Among enrolled patients, seven showed AHI > 10 (mean ± SD: 17.8 ± 6.3) and were included in group one (severe OSAS grade), whereas 11 patients showed AHI ≤ 10 (mean ± SD: 6.3 ± 2.6) and were included in group two (mild/moderate OSAS grade).

ADRB2 genotypes

The allelic distribution of the selected SNPs adhered to Hardy-Weinberg equilibrium (data not shown).

We found that five out of seven patients (86%) with an AHI > 10 showed the rs1042713 G/G genotype, while two out of seven patients (29%) had a G/A genotype, and no patients carried an A/A genotype. In patients with AHI ≤ 10, only one patient out of 11 (9%) carried the rs1042713 G/G genotype, while three patients (27%) carried a G/A genotype, and seven patients (64%) carried an A/A genotype. Using a χ² test for trend, we found that the rs1042713 G/G genotype was significantly more frequent in patients with AHI > 10 compared to those with AHI ≤ 10 (P = 0.002). This result was confirmed with Fisher's exact test using a recessive model (P = 0.012). In contrast, the allelic frequencies of both the rs1042714 and rs1800888 SNPs were not significantly different between the two groups of subjects (Table 2).

**Table 2:** ADRB2 SNPs frequency in enrolled patient ^*^: χ^2^-test for trend; ^#^: Fisher Exact test n: number of patients; nd: not determinable; SNPs: single nucleotide polymorphisms; OR: odds ratio

SNP	Genotype	AHI > 10 n (%)	AHI ≤ 10 n (%)	P-value^*^	P-value^#^	OR (95% CI)
rs1042713	G/G	5 (71)	1 (9)	0.002	0.012	25.0 (1.1-511.0)
	A/G	2 (29)	3 (27)			
	A/A	0 (0)	7 (64)			
rs1042714	C/C	1 (14)	3 (27)	0.675	1.000	0.93 (0.11-7.7)
	C/G	4 (57)	5 (46)			
	G/G	2 (29)	3 (27)			
rs1800888	C/C	4 (71)	11 (100)	nd	0.0858	0.06 (0.002-1.31)
	C/T	3 (29)	0 (0.0)			
	T/T	0 (0.0)	0 (0.0)			

This result was confirmed by the observation that the AHI was significantly higher in subjects homozygous for the G allele of the rs1042713 SNP compared to those homozygous for the A allele, while no correlations were found between the rs1042714 and rs1800888 SNPs and AHI score (Table 3).

**Table 3:** Correlation between ADRB2 SNPs frequency and clinical parameters in enrolled patients IL: interleukin; CRP: C-reactive protein; ESR: erythrocyte sedimentation rate; SNPs: single nucleotide polymorphisms; AHI: apnea-hypopnea index *: P-value < 0.01 vs A/A (one-way analysis of variance with Bonferroni’s multiple comparison test).

Parameters	SNPs								
	rs1042713			rs1042714			rs1800888		
	G/G	G/A	A/A	C/C	C/G	G/G	C/C	C/T	T/T
AHI (mean ± SD)	17.1 ± 7.0*	11.3 ± 3.9	4.9 ± 2.0	10.6 ± 12.4	11.7 ± 6.0	7.4 ± 4.7	6.8 ± 4.8	10.1 ± 5.8	-
IL-6 (mean ± SD - pg/ml)	0.1 ± 0.2	0.6 ± 0.8	0.4 ± 0.4	0.3 ± 0.5	0.2 ± 0.2	0.5 ± 0.6	0.2 ± 0.4	0.8 ± 0.7	-
IL-8 (mean ± SD - pg/ml)	5.3 ± 0.3	5.3 ± 0.3	5.3 ± 0.3	5.4 ± 0.5	5.3 ± 0.3	5.2 ± 0.3	5.3 ± 0.1	5.2 ± 0.5	-
Neutrophils (mean ± SD ×10^3^/μL)	3.2 ± 0.6	3.3 ± 0.7	3.1 ± 1.3	3.2 ± 2.5	3.1 ± 0.9	3.3 ± 0.5	2.9 ± 1.3	3.7 ± 0.3	-
Eosinophils (mean ± SD ×10^3^/μL)	0.6 ± 0.5	0.3 ± 0.3	0.6 ± 0.4	0.5 ± 0.4	0.6 ± 0.5	0.5 ± 0.2	0.5 ± 0.4	0.9 ± 0.5	-
CRP (mean ± SD - mg/L)	1.5 ± 2.2	0.3 ± 0.1	1.3 ± 1.4	2.9 ± 2.7	0.5 ± 0.2	1.3 ± 1.6	0.6 ± 0.3	0.4 ± 0.2	-
ESR (mean ± SD - mm/h)	8.7 ± 2.1	2.7 ± 1.2	5.5 ± 2.5	7.0 ± 1.4	6.2 ± 4.1	3.7 ± 1.2	4.3 ± 2.1	7.3 ± 3.5	-

Finally, we did not find a significant association between patients' genetic profiles and either the inflammatory parameters (Table 3) or ADRB2 mRNA expression levels in PMN (Table 4).

**Table 4:** Correlation between ADRB2 SNPs and ADRB2 mRNA expression levels in circulating PMN ^*^: one-way analysis of variance with Bonferroni's multiple comparison; ^#^: Student t-test PMN: polymorphonuclear neutrophil; SNPs: single nucleotide polymorphisms

SNPs		ADRB2 mRNA expression levels	P-value
rs1042713	G/G	1.45 x 10^-8^± 7.36 x 10^-10^	0.3953^*^
	A/G	1.41 x 10^-8^ ± 1.5 x 10^-9^	
	A/A	1.39 x 10^-8^ ± 8.33 x 10^-10^	
rs1042714	C/C	1.38 x 10^-8^ ± 9.18 x 10^-10^	0.1220^*^
	C/G	1.45 x 10^-8^ ± 1.15 x 10^-9^	
	G/G	1.38 x 10^-8^ ± 7.36 x 10^-10^	
rs1800888	C/C	1.41 x 10^-8^ ± 1.01 x 10^-9^	0.4137^#^
	C/T	1.44 x 10^-8^ ± 1.16 x 10^-9^	
	T/T	-	

## Discussion

The main result of this study is that, in a cohort of Caucasian pediatric patients, the rs1042713 SNP (G/G genotype) in ADRB2 was more frequent in patients with severe OSAS grade compared to patients showing mild/moderate OSAS grade.

In pediatric patients, OSAS severity is closely linked to the development of various morbidities, including behavioral problems, learning difficulties, cardiovascular complications, and growth retardation [2,3], as well as treatment approaches that range from lifestyle modifications to surgery [24]. OSAS severity grade is influenced by both non-genetic (sleep-wake cycle, endothelial function, fat distribution, ventilation control, craniofacial morphology, and upper airway muscle tone) and genetic factors. Genetic factors are estimated to account for 35-40% of the variance in OSAS severity, and some studies have suggested links between a patient's genetic profile and OSAS-related complications [25-28], such as disease susceptibility [28,29] and disease severity in obese individuals [30]. Present findings, which report the association between the rs2071943 SNP in ADRB2 and OSAS severity in pediatric patients, are then consistent with previous observations in this field.

The findings reported in this study open the possibility of considering the rs1042713 SNP as a potential predictive marker for OSAS severity. However, only after our findings are confirmed in a prospective study with a larger sample size do we move on to the next phase, in which the identification of SNPs in the gene encoding the ADRB2 receptor could contribute (alongside currently available non-genetic markers) to selecting appropriate OSAS management.

Even more intriguing are the potential roles that SNPs in ADRB2 could play in the pathogenesis of OSAS. Indeed, it is well known that the sympathetic nervous system represents an important element in the modulation of immune system functions, including inflammation [10,31]. In particular, ADRB2 exerts significant anti-inflammatory effects, such as reducing PMN migration, decreasing the production of ROS and IL-8 [11], and inhibiting extracellular trap formation [5]. In our recent study, we observed a correlation between OSAS symptom improvement following adenotonsillectomy and the patient's inflammatory status normalization and reduction in ADRB2 mRNA expression levels in PMN [12]. While these findings may initially appear inconsistent with the previously documented anti-inflammatory effects attributed to ADRB2 [5,11], it is plausible to hypothesize that the inhibitory effects of ADRB2 on inflammatory processes become more pronounced during heightened inflammatory conditions and diminish when inflammation returns to baseline levels. The present findings suggest that the G/G genotype in the rs1042713 SNP may contribute to OSAS severity by diminishing the inhibitory effects of this receptor on inflammatory processes, a key factor in OSAS pathogenesis and progression [3,4,6]. Our working hypothesis is that the G/G genotype, known to reduce ADRB2 membrane density, increases the likelihood that circulating CTs, which are elevated in OSAS patients [7], further reduce ADRB2 expression. The resulting decrease in ADRB2 expression on cell membranes may weaken the anti-inflammatory effects exerted by this receptor [5,10,11], thereby worsening OSAS severity. Of course, this hypothesis must be confirmed in further studies conducted on larger cohorts of pediatric patients, where the patient's inflammatory profiles, ADRB2 expression levels, and genetic profiles are carefully evaluated.

The main limitation of this study is the small number of enrolled patients. However, it is important to note that we focused on a very young pediatric population with stringent selection criteria. These rigorous selection criteria are the real strength of the study, ensuring population homogeneity, reducing confounding factors, and maximizing the likelihood that observed differences in OSAS severity grade were due to genetic differences rather than other non-genetic factors.

## Conclusions

In conclusion, the present results allow us to identify, for the first time in pediatric OSAS patients, a correlation between disease severity and rs1042713 SNP in ADRB2. This finding, if confirmed in further studies including a greater number of subjects, could become a useful tool in clinical practice by providing a biomarker for the early identification of patients at high risk of developing severe OSAS. Moreover, the possible relationship between the patient's genetic profile and the inflammatory process in OSAS could open the way for identifying patient-oriented therapeutic strategies, which may include personalized disease treatment, reducing morbidity and mortality associated with the OSAS syndrome.
